# Knowledge and perceptions of South African blood donors towards biobanking and stool donation

**DOI:** 10.4102/sajid.v39i1.645

**Published:** 2024-10-31

**Authors:** Shantelle Claassen-Weitz, Elloise du Toit, Sugnet Gardner-Lubbe, Brian Kullin, Gregory Bellairs, Caroline Hilton, Anika Chicken, Kirsten Welp, Hannah Livingstone, Adrian Brink

**Affiliations:** 1 Department of Pathology, Faculty of Health Sciences, University of Cape Town, Cape Town, South Africa; 2 Department of Statistics and Actuarial Sciences, Faculty of Economic and Management Sciences, Stellenbosch University, Stellenbosch, South Africa; 3 Western Cape Blood Services, Cape Town, South Africa; 4 National Health Laboratory Service, Groote Schuur Hospital, Cape Town, South Africa; 5 Institute of Infectious Disease and Molecular Medicine, Faculty of Health Sciences, University of Cape Town, Cape Town, South Africa

**Keywords:** blood donations clinics, human microbiota, *Clostridioides difficile*, faecal microbiota transplantation, microbial dysbiosis, stool donors, microbiome biobank

## Abstract

**Background:**

The complexity of contexts and varied purposes for which biome donation are requested are unknown in South Africa.

**Objectives:**

The aim of this study was to provide strategic data towards actualisation of whether a stool donor bank may be established as a collaborative between Western Cape Blood Services (WCBS) and the University of Cape Town (UCT).

**Method:**

We designed a cross-sectional, questionnaire-based survey to determine willingness of WCBS blood donors to donate stool specimens for microbiome biobanking. The study was conducted between 01 June 2022 and 01 July 2022 at three WCBS donation centres in Cape Town, South Africa. Anonymous blood donors who met the inclusion criteria were enrolled. Anonymised demographic and interview data were analysed statistically.

**Results:**

Analysis of responses from 209/231 blood donors demonstrated in a logistic regression model that compensation (*p* < 0.001) and ‘societal benefit outweighs inconvenience’ beliefs (*p* = 7.751e-05) were covariates significantly associated with willingness to donate stool. Age was borderline significant at a 5% level (*p* = 0.0556). Most willing stool donors indicated that donating stool samples would not affect blood donations (140/157, 90%). Factors decreasing willingness to donate were stool collection being unpleasant or embarrassing.

**Conclusion:**

The survey provides strategic data for the establishment of a stool bank and provided an understanding of the underlying determinants regarding becoming potential donors.

**Contribution:**

This is the first report on the perspectives of potential participants in donating samples towards a stool microbiome biobank in South Africa, a necessity for faecal microbiota transplantation (FMT).

## Introduction

The human body is home to an array of microorganisms including bacteria, viruses, fungi, archaea and even protists, collectively termed human microbiota.^1^ Imbalances in compositional and/or functional ecology of these microbial communities, referred to as microbial dysbiosis, have been associated with inflammatory and infectious diseases across different body sites.^2^

The largest component of the human microbiota can be found in the gastrointestinal tract (GIT), where it plays a crucial role in the modulation of several host functions including nutrient absorption,^3^ immune modulation and protection against pathogens.^4,5^ Changes in the symbiotic relationship between the microbiota and the GIT microenvironment, constituting immune cells and enteric neurons, trigger chronic diseases including GIT inflammation-related disorders, inflammatory autoimmune disorders and cardiometabolic disorders.^6^ Gastrointestinal tract microbial dysbiosis also causes diarrhoea via the overgrowth of enteric pathogens such as *Clostridioides difficile* – the primary cause of antibiotic-associated diarrhoea worldwide.^7^ To date, several therapeutic strategies aimed at restoring the GIT ecosystem have been implemented. Faecal microbiota transplantation (FMT) is one such therapy that has increasingly been adopted by healthcare centres globally to treat recurrent *C. difficile* infections.^8,9,10,11^ Faecal microbiota transplantation is also being investigated as a treatment modality for other infectious and inflammatory conditions associated with dysbiotic microbial communities including inflammatory bowel diseases (IBD), irritable bowel syndrome (IBS), metabolic syndrome, obesity, malnutrition, autoimmune diseases and autism spectrum disorders.^12^ More studies are needed to better understand mechanisms of interactions between microbes and their host to modulate structural and functional characteristics for improving health.^13^

Globally, microbiome biobanks are required for extensive microbiome research and the development of targeted therapeutics. There is no established national microbiome biobank such as Open Biome^14^ in South Africa.^15,16^ Given that blood donors have been identified to constitute an ideal cohort for microbiome collections,^17,18^ and that several microbiome biobanks have been established in collaboration with blood donor services in Europe and the United Kingdom, this study aims to investigate interest and willingness among blood donors in the Western Cape, South Africa, in becoming microbiome donors. Biobanking in South Africa is hindered by a myriad of complex societal considerations and ethico-legal challenges; however, specific data on willingness for various biobanking are not available.^19^

The primary aim of our study is to provide strategic data for all stakeholders towards actualisation of whether a biobank of stool microbiomes may be established for the purpose of FMT, as a collaboration between the Western Cape Blood Services (WCBS) and the University of Cape Town (UCT). The secondary aim was to concurrently ascertain the social context, knowledge and attitude of potential participation in establishing a stool microbiome biobank.

## Research methods and design

### Study design

We designed a cross-sectional, questionnaire-based survey to determine willingness of WCBS blood donors to donate stool specimens for microbiome biobanking.

### Setting

This study was conducted between 01 June 2022 and 01 July 2022 at three WCBS donation centres in Cape Town, South Africa spanning a total area of 106 km^2^.

### Study population and sampling strategy

Anonymous blood donors who met the inclusion criteria of being between the ages of 18 and 50 years and willing to provide informed consent were interviewed. The upper age limit of 50 years was chosen because of the gut microbiome’s changing nature with age and its subsequent decline in diversity. Infographics on stool donation were shared and included: (1) reasons for donating stool specimens for microbiome biobanking, (2) donor eligibility and (3) specimen collection approaches (A GutAlive stool collection kit^20^ was demonstrated to blood donors).

### Data collection

After obtaining informed consent, we collected respondents’ demographics (age, gender and occupation) via pre-populated questionnaires. The questionnaires also collected information on respondents’ history of blood and organ donation, FMT-related knowledge and perceptions and modifiable aspects of stool specimen donations. We also collected information on the primary reasons for becoming or not becoming a stool specimen donor. We approached donors eligible for blood donations following the donation process. Ineligible blood donors were approached following the screening process. Compensation was not offered to participants in the survey and ethical approval was obtained before study commencement.

### Data analysis

Anonymised demographic and interview data were aggregated for descriptive purposes and statistical analysis. Data from each questionnaire were captured in the database and confirmed by two co-investigators.

The variables considered in this investigation were divided into three categories. Firstly, the variable ‘willing donor’ (participants who had indicated their willingness to donate stool for FMT) was modelled as a function of the possible covariates (participant characteristics) listed in Online Appendix 1 Table 1. Secondly, the variables pertaining specifically to willing donors were used to characterise the willing donors (Online Appendix 1 Table 2). Thirdly, the reasons for being unwilling to donate stool were investigated (Online Appendix 1 Table 3). Participants who provided no informed consent, participants < 18 and > 50 years of age and participants who provided incomplete questionnaires were excluded from the study. In addition, records where the ‘willing donor’ field was missing were excluded.

The random Forest package^21^ in R software was used to identify variables that contribute to being a ‘willing donor’. A total of 500 classifications trees were built using random subsets of the covariates. Based on all 500 trees, a variable importance plot (VIP) was produced, indicating the importance of each covariate in classifying potential donors as willing or not. A logistic regression model was fitted for variables showing the largest mean decrease in accuracy. Variables were selected in a stepwise manner: (1) remove the variable with largest mean decrease in accuracy in the VIP and add it to a logistic regression model; (2) fit another random forest to produce a VIP and (3) repeat until variables added to the logistic regression are not significant for classification.

A multivariate analysis was performed to further investigate variables used to characterise willing stool donors. The latter allowed to determine which response to a particular question more often corresponded to responses to other questions. A joint correspondence analysis was performed using the ca package^22^ in R software. Subset correspondence analysis was selected to suppress the use of the ‘missing’ categories in determining the plot while keeping the row totals constant. Proportions (indicated as pie slices in the plot) were computed based on the frequency of responses for each category in a specific variable.

### Ethical considerations

Ethical clearance to conduct this study was obtained from the Human Research Ethics Committee (HREC), University of Cape Town (No. HREC122/2022). The relevant guidelines and regulations were followed during the performance of this survey. All participants provided informed, written consent for enrolment in the study.

## Results

A questionnaire was given out to blood donors visiting one of three blood donations clinics in Cape Town, South Africa. A total of 231 blood donors took part in the study. Twelve participants were < 18 and > 50 years of age, and four participants provided incomplete questionnaires and were excluded from the study. Of the 215 remaining participants, an additional six records where the ‘willing donor’ field was missing were excluded. Overall, responses from 209 participants were included in the analysis (Table 1).

**TABLE 1:** Participant characteristics of blood donors surveyed as potential stool donors (*N* = 209).

Participant characteristics	*n*	%
**Site at which questionnaire was collected:**		
Blue Route Mall	51	25
Long Street	84	40
N1 City Mall	74	35
**Participant gender:**		
Female	120	57
Male	88	42
Other	1	1
**Participant age at visit (years):**		
18–21	28	13
21–30	66	32
31–40	60	29
41–50	55	26
**Categorical classification of participant occupation:**		
Learner/student	41	20
Employed	153	73
Unemployed	10	5
Missing	5	2
**Do you regularly donate blood?**		
Yes	159	76
No	50	24
**Are you an organ donor/would you consider becoming an organ donor?**		
Yes	113	54
No	63	30
Unsure	33	16
**Do you have prior knowledge of the concept of a healthy stool microbiome?**		
Yes	60	29
No	131	63
Unsure	18	8
**Do you have prior knowledge of what an FMT is?**		
Yes	34	16
No	159	76
Unsure	16	8
**Do you have prior knowledge of how FMTs could help patients?**		
Yes	35	17
No	153	73
Unsure	21	10
**Do you have prior knowledge of what a stool collection kit looks like?**		
Yes	32	15
No	175	84
Missing	2	1
**Would you consider being a stool sample donor if receiving the following compensation per donation:**		
None	15	7
≤ ;R150.00 (≤ ;$8.00)	48	23
> ;R150.00 to ≤ ;R250.00 (> ;$8.00 to ≤ ;$13.00)	68	33
> ;R250.00 (> ;$13.00)	9	4
Any amount/travel costs	10	5
Missing	59	28
**Do you believe helping other is more important than any inconvenience being a stool donor may impose?**		
Yes	183	88
No	19	9
Missing	7	3

FMT, Faecal microbiota transplant.

### Participant characteristics

Most participants included in the study were willing to donate stool (159/209, 76%). More females participated in the study compared to males (Table 1). Participants were primarily between the ages of 21 and 40 (126/209, 61%), and most participants were employed (153/209, 73%). Most participants did not have prior knowledge of the concept of a healthy stool microbiome (131/209, 63%), FMT (159/209, 76%), how FMTs could help patients (153/209, 73%) and what a stool collection kit looked like (175/209, 84%) (Table 1). Most participants indicated that they would become a stool sample donor even if they were not economically compensated or not by very much (> 0 to ≤ 250 South African rand [ZAR]) or any amount/travel costs as economic compensation (131/209, 63%). Larger amounts (> 250 ZAR) were not necessary to further incentivise stool donation (9/209, 4%) (Table 1). Almost one third of participants did not provide information regarding economic compensation. Most participants indicated that helping others would outweigh any inconvenience stool donation may impose (183/209, 88%).

### Willingness to become a stool donor by participant characteristics: Identifying variables that contribute to being a ‘willing donor’

**FIGURE 1:**
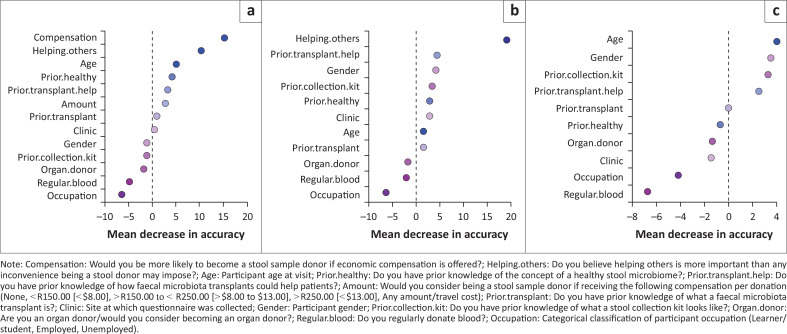
Variance importance plots showing important covariates in accurately classifying potential donors as a willing donor. (a) The largest mean decrease in accuracy was found with the removal of compensation, showing that compensation plays the most important role in accurately classifying potential donors as willing or not. Removing compensation (b) or both compensation and opinions as to whether helping others outweighs the inconvenience of donation (c) from the data lead to different results when applying the random forest method, confirming that the results are obtained by chance. Some of the covariates that appeared as important are prior knowledge of how faecal microbiota transplants (FMTs) could help the others, gender and prior knowledge of what a stool collection kit looks like.

Variance importance plots identified compensation, followed by beliefs as to whether helping others would outweigh the inconvenience of stool donation (‘benefit outweighs inconvenience’), and age, as the variables with the most important role in accurately classifying potential donors as willing or not (Figure 1A). Other important covariates were prior knowledge of how FMTs specifically could help others, gender and prior knowledge of what a stool collection kit looks like (Figure 1A–C). Fitting logistic regression models showed that prior knowledge of how FMTs could help others; gender or prior knowledge of what a stool collection kit looks like were not significant factors in determining willingness to donate.

A logistic regression model with compensation, ‘benefit outweighs inconvenience’ beliefs and age showed that compensation (*p* < 0.001) and ‘benefit outweighs inconvenience’ beliefs (*p* < 0.001) were covariates significantly associated with willingness to donate stool. Participants were 29% more likely to donate stool if compensated (odds ratio: 1.294; CI 1.158, 1.446). Participants who felt that helping others was more important than any inconvenience being a stool donor may impose were 43% more likely (CI 1.185, 1.727) to be willing stool donors. Age bordered on significant at a 5% level (*p* = 0.0556). Coefficients suggest that there was a larger likelihood of being a donor, the older the participant (21–30 years: –0.001, 31–40 years: 0.013, 41–50 years: 0.181). Including compensation, a belief that potential benefit to donors outweighs donation inconvenience and age in a logistic regression model allowed for 79% of participants to be classified correctly as willing stool donors.

### Characteristics of willing stool donors

**FIGURE 2:**
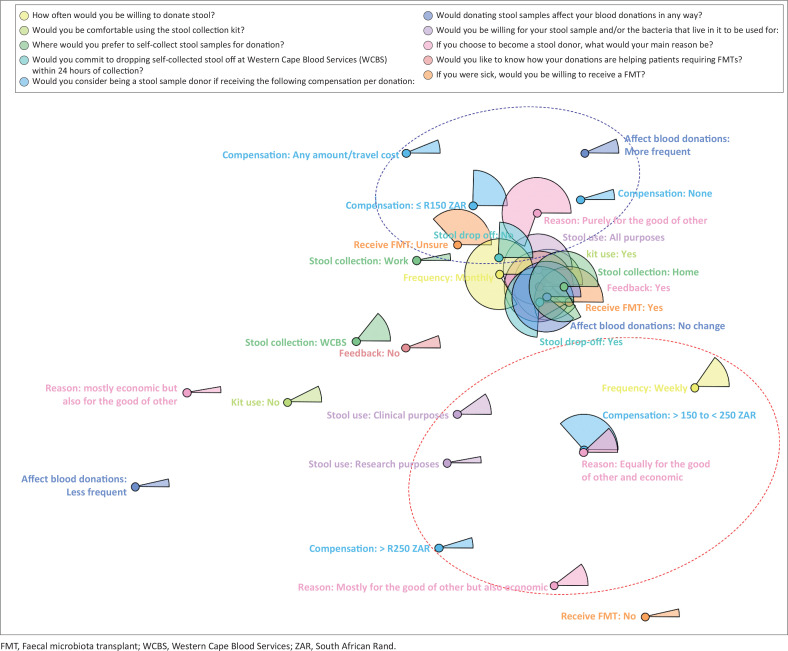
Multivariate analysis of variables used to characterise willing stool donors. Each variable (and its categories) used to characterise willing stool donors is represented using a unique colour. The frequency of responses for each category in a specific variable are shown as proportions (represented as pie slices in the plot). Category levels that appear close by, tend to appear together in responses while category levels that appear far apart, typically belong to different participants.

Of the 159 willing stool donors, two participants indicated ‘never’ when asked ‘How often would you be willing to donate a stool sample?’ and ‘none of the above’ when asked ‘Would you be willing for your stool sample and/or the bacteria that live in it to be used for…?’ These two participants were excluded from downstream analyses (Table 2). The joint correspondence analysis showed that most willing stool donors agree on using the stool collection kit (140/157, 89%), to self-collect at home (130/157, 83%), as opposed to work or WCBS clinics and could commit to dropping off stool within 24 h of collection (117/157, 75%) (Table 2; Figure 2). Most willing stool donors would be willing to donate stool monthly (131/157, 83%). Willing stool donors primarily agreed that donating stool samples would not interfere with blood donation frequencies (140/157, 90%) and that they would donate purely for the good of others, but compensation (< R250.00) would increase the likelihood of donation (63%), while 22% omitted this information (Table 2). More than 90% of willing stool donors would like feedback on how their donations helped patients and indicated that stool could be used for both clinical and research purposes (136/157, 87%). (Table 2 and Figure 2). Ninety-two willing stool donors (61%) expressed a willingness to receive a FMT if needed; however, 38% were unsure (Table 2). As willing stool donors made up approximately 75% of participants overall (157/209) and as a group provided 75% or more of participant responses to most questions (Figure 2), we can estimate that approximately 60% of all participants share these views.

**TABLE 2:** Participant characteristics of willing stool donors (***N*** = 157).

Participant characteristics	*n*	%
**How often would you be willing to donate stool?**		
Weekly	23	15
Monthly	131	83
Missing	3	2
**Would you be comfortable using the stool collection kit?**		
Yes	140	89
No	12	8
Missing	5	3
**Where would you prefer to self-collect stool samples for donation?**		
Home	130	83
WCBS	22	14
Work	5	3
**Would you be able to commit to dropping self-collected stool off at the WCBS HQ, Pinelands, Cape Town within 24 h of collection?**		
Yes	117	75
No	38	24
Missing	2	1
**Would you consider being a stool sample donor if receiving the following compensation per donation:**		
None	8	5
≤ R150.00 (≤ $8.00)	39	25
> R150.00 to ≤ R250.00 (> $8.00 to ≤ $13.00)	59	38
> R250.00 (> $18.00)	8	5
Any amount/travel costs	9	6
Missing	34	22
**Would donating stool samples affect your blood donations in any way?**		
No changes to blood donations	141	90
More frequent blood donations	11	7
Less frequent blood donations	5	3
**Would you be willing for your stool sample and/or the bacteria that live in it to be used for:**		
Clinical purposes	14	9
Research purposes	5	3
All the above	136	87
Missing	2	1
**If you choose to become a stool donor, what would your main reason be?**		
Purely for the good of others	110	70
Mostly for the good of others but also economic	17	11
Equally for the good of others and economic	21	13
Mostly economic but also for the good of others	5	3
Missing	4	3
**Would you like to know how your donations are helping patients requiring FMTs?**		
Yes	143	91
No	11	7
Missing	3	2
**If you were sick, would you be willing to receive a FMT?**		
Yes	92	59
Unsure	59	38
No	6	3

WCBS, Western Cape Blood Service; FMT, Faecal microbiota transplant; HQ, headquarters.

Willing stool donors who would consider being a donor if they received larger amounts of compensation (> R150.00), would donate stool more frequently (weekly intervals), would donate for the good of other and economic reasons and would want stool to be used either only for research or for clinical use (Figure 2). Conversely, willing stool donors who would consider being a donor for smaller amounts (≤ R150.00) or no compensation tended to want to donate purely for the good of others. The latter donors appeared on the same side of the plot as willing stool donors who do not commit to dropping off samples within 24 h and those unsure about receiving an FMT if needed (Figure 2).

### Reasons for being unwilling to donate stool

Each of the unwilling stool donors (*N* = 50) provided one or more reasons for being unwilling to donate stool. The most important reasons provided were that stool collection would be unpleasant (31/86, 36%) and embarrassing (18/86, 21%). This was followed by the fact that regular (monthly: 12% and weekly: 10%) stool donation would be too much of a commitment and logistics (7%). Few unwilling stool donors indicated that medical examinations at the WCBS clinic during donations would be too time consuming and exhaustive (6%), that the collection procedure would be too complicated (2%) or that stool collection would not align with cultural beliefs (5%). A single unwilling stool donor did not agree with the concept of FMT procedures.

Unwilling stool donors primarily indicated that they were unsure or unwilling to receive an FMT if needed compared to willing stool donors who primarily indicated that they would be willing to receive an FMT.

## Discussion

The concept of stool donation and FMT is mostly unknown within the South African population, as its use in the clinical field is relatively new and is not common knowledge yet. It was therefore surprising that most respondents in Cape Town (76%) said they would be willing to become stool donors. Once the idea of a healthy microbiome and the benefits that an FMT could offer a patient in need were explained to the blood donor, those willing to donate their stool said that they would do so to help others (i.e. the patient), especially if financial compensation was offered, with ≤ R150.00 (≤ $8.00) being sufficient. This agrees with previous reports from high-income countries where compensation was found to increase intent to become a stool donor.^23^ Our study also highlighted age as a possible determining factor, with older participants being more likely to donate stool than younger ones. Conversely, a multicentre study conducted in three high-income countries (Canada, United Kingdom and United States) reported that intended participation was not associated with age,^23^ suggesting that using age as a variable for identifying willing stool donors should be considered on a case-by-case basis.

Every aspect of stool donation, from collection, donation frequency and sample delivery should be made as easy and pleasant as possible. Eligible blood donors indicated that stool collection would be unpleasant (36%) and embarrassing (21%). This is a common theme^24^ and overcoming it is challenging, as the general perception of stool is unpleasant. Although the GutAlive stool collection kit^20^ offers a relatively clean collection process, it does not altogether remove the potentially unpleasant nature of collecting stool. Financial compensation may help outweigh the unpleasant nature of stool sample collection, as shown in this cross-sectional study, where compensation was the largest factor in determining willingness to donate. In fact, compensation boosted the likelihood of donation by 29%. Compensation was closely followed by helping others, with 43% of respondents being willing to donate stool, as helping another person outweighed the inconvenience of stool collection. Thus, promoting the fact that becoming a stool donor could help save a life^25^ would clearly be a strong motivator, given the already altruistic nature of blood donors. Further, exposure therapy to normalise stool collection^26^ may also be a viable option. If age is to be considered, its specific effect on the outcome could be utilised to focus recruitment efforts on those participants who are more likely to donate stool (in this study, this was the older participants).

This cross-sectional study has been important in highlighting the possible ambivalence and reasons thereof, in participating in a stool biobank. This will allow us to address and resolve these concerns so that donors feel more confident to become stool donors. Building confidence in donors will involve further communication via educational campaigns and advertising detailing the process of stool collection from start to finish and also sharing previous donors’ testimonies. This would raise awareness and increase familiarity with the process. Given that the costs involved in screening potential stool donors to become continuous donors is significant, it is important that participants are ‘screened’ to ensure their eligibility and sustained participation in building a feasible process to start a stool biobank for the use of FMT in South Africa.

Our study had several limitations. Our sample size was small and hence drawing any major conclusions from the results would not produce a robust report. The study was conducted at three WCBS donor sites in Cape Town and is thus not representative of the rest of the Western Cape or South Africa as a whole.

## Conclusion

To conclude, our data have indicated that the City of Cape Town in the Western Cape, South Africa, is a feasible option to start a stool biobank, with most blood donors being open to the concept. We will need to extend the investigation further into South Africa to confirm that willingness to participate in a stool biobank is the same in other areas of the country. In addition, doing this in collaboration with the WCBS provides an accessible and sustainable source of potential donors to meet the continuous needs of the future stool biobank.
